# Supplementary material for: ERα-associated translocations underlie oncogene amplifications in breast cancer
Source: Nature. 2023 May 17;618(7967):1024–32. doi: 10.1038/s41586-023-06057-w (PMC10307628; doi:10.1038/s41586-023-06057-w)
Supplement: Supplementary file 1 — Additional discussions including Supplementary Fig. 1–8 and references. [file 41586_2023_6057_MOESM1_ESM.docx]

**Supplementary Note**

Lee *et al*. ERɑ-associated translocations underlie oncogene

amplifications in breast cancer, *Nature*, 2023

Benchmark analysis comparing the HMF pipeline vs. the PCAWG pipeline 2

In-house filter for L1-mediated transductions 3

Putative oncogenes in the focally amplified regions 3

Mutational signature at the vicinity of the rearrangement breakpoints 6

A male breast cancer case with translocation-bridge amplification 7

Fusion gene analysis 9

Mechanisms of DNA breaks in *SHANK2* and *TENM4* loci 10

Role of physical proximity in early translocations 12

Supplementary note references 13

**Benchmark analysis comparing the HMF pipeline vs. the PCAWG pipeline**

We ran the Hartwig Medical Foundation (HMF) pipeline for the 208 breast cancer cases in the PCAWG cohort and compared the output to the PCAWG consensus variant calls based on the joint-calling strategy^1^.


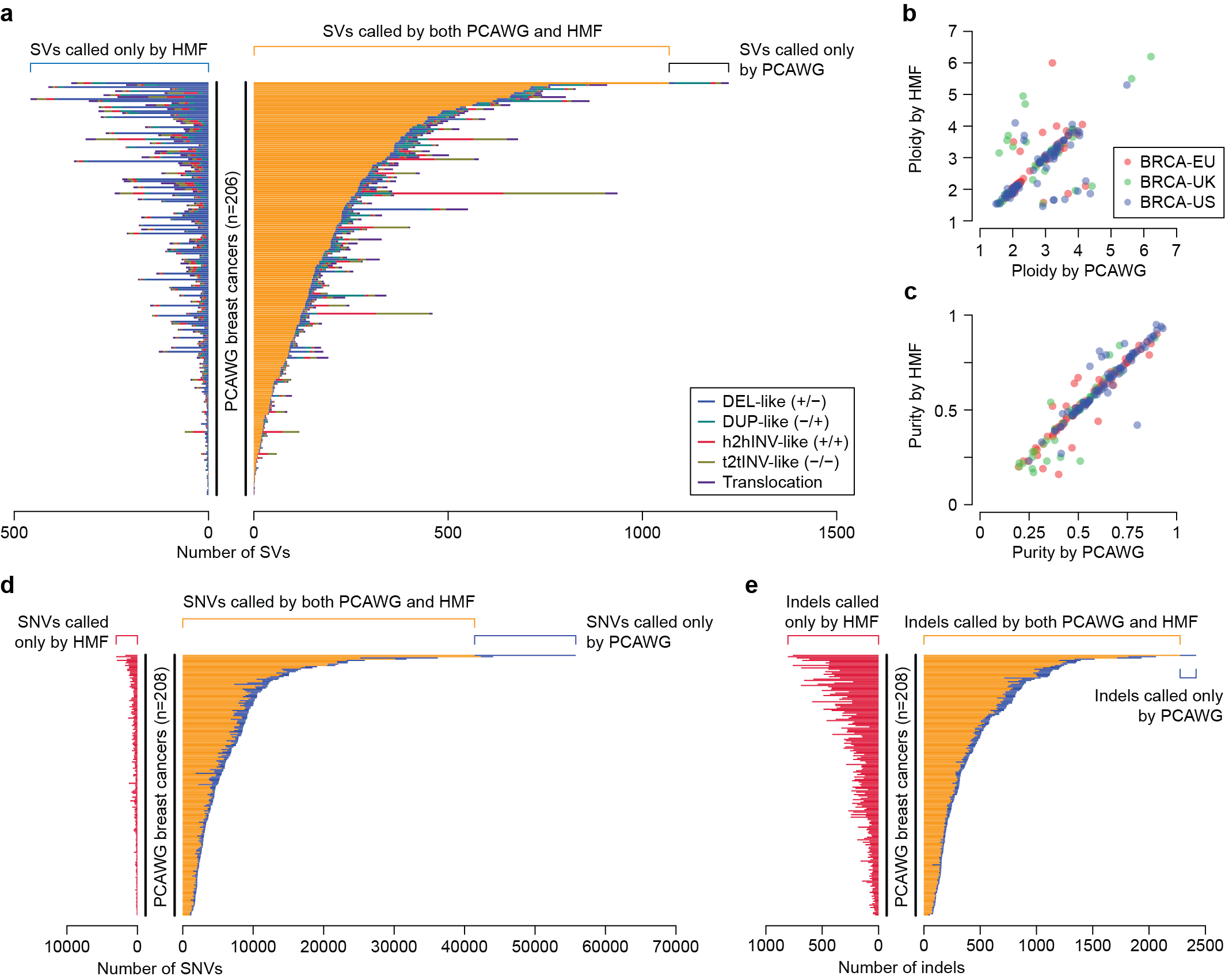


**Supplementary Fig. 1. Comparison of variant calls between the HMF-based and the PCAWG bioinformatic pipelines in the PCAWG breast cancers.**

Comparison of **a,** structural variations (SVs), **b,** tumor ploidy estimates, **c,** purity estimates, **d,** single nucleotide variants (SNVs), and **e,** small indels. Among the 208 breast cancer cases in the PCAWG cohort, SVs were identified in 206 cases by the PCAWG study.

The HMF pipeline detected 39,442 out of 49,404 SVs in the PCAWG consensus calls (recovery rate = 79.8%; **Supplementary Fig. 1a**). Among the SVs that were missed by HMF but were identified by PCAWG, some were short, non-reciprocal, and singleton inversions, which were likely artifactual. We found several TCGA samples that were heavily affected by these inversions in the PCAWG analysis (e.g., 548 in TCGA-A2-A04P, 289 in TCGA-AO-A0J4, and 219 in TCGA-A8-A07I). Most of these inversions were supported by two SV callers, dRanger and SvABA, in the PCAWG joint calling pipeline. The HMF pipeline also captured a similar pattern of short, non-reciprocal inversions in these samples, albeit less frequently than by the PCAWG pipeline. Therefore, for the 11 TCGA samples in which we found large number of SVs (≥200) with a high inversion rate (≥0.6), we applied a custom filter to exclude the singleton, non-reciprocal inversions <10Kbp in size, with a quality score <1100. The HMF pipeline also identified 14,640 SVs that were missed by PCAWG. A large fraction of them (n=5,947; 40.6%) were small SVs (≤100 bp), which were not called as SVs in the PCAWG pipeline (where an SV was defined as >100 bp).

The ploidy and purity estimates were also largely concordant between the HMF and the PCAWG pipelines (**Supplementary Fig. 1b, c**). For SNVs and indels, the HMF pipeline detected 1,237,872 of 1,447,162 PCAWG SNVs (recovery rate = 85.5%; **Supplementary Fig. 1d**) and 82,391of 92,784 PCAWG indels (88.8%; **Supplementary Fig. 1e**). Based on this excellent concordance, we applied the HMF pipeline to the entire cohort. Among the 782 cases processed in the pipeline, two were excluded due to poor data quality (PD4956 in the Sanger study and IBC111 in the Yale study). Therefore, 780 breast cancer cases were included in the final analysis.

**In-house filter for L1-mediated transductions**

We performed a visual inspection of 780 breast cancer cases to verify the concordance between the copy number junctions and the SVs. We found that several cases had a large number of inter-chromosomal translocations to multiple different regions emanating from a single genomic locus without causing large-scale copy number alterations in the neighborhood. These events involved several genomic regions, most commonly a locus in 22q (around chr22:29,065,741). These observations were consistent with L1-mediated transductions. Given that these transduction events could affect the statistics regarding inter-chromosomal translocations, we systematically analyzed L1 transductions in 279 breast cancers (PCAWG + Ferrari *et al*.^19^) using xTea^55^, a method we have developed for transposable element detection. Based on the result, we identified 18 hot source retroelements in breast cancer (**Supplementary Table 4**) and filtered the SVs overlapping with these regions in the 780 breast cancers.

**Putative oncogenes in focally amplified regions**

We used GISTIC2 (v2.0.23) to identify significantly amplified or deleted genomic regions in 780 breast cancer genomes (**Supplementary Fig. 2a**). Oncogenes and tumor suppressors were frequently located in the peaks of amplifications and the valleys of deletions, respectively. After statistical filtering and merging the neighboring peaks, we focused on the nine most recurrent amplification peaks (**Supplementary Fig. 2b**). We analyzed these peaks by calculating the frequency of amplified regions (≥4x of the ploidy estimate of the given tumor) across samples (rather than using the absolute copy number, to limit the impact of the tumors showing extreme copy-number amplifications).


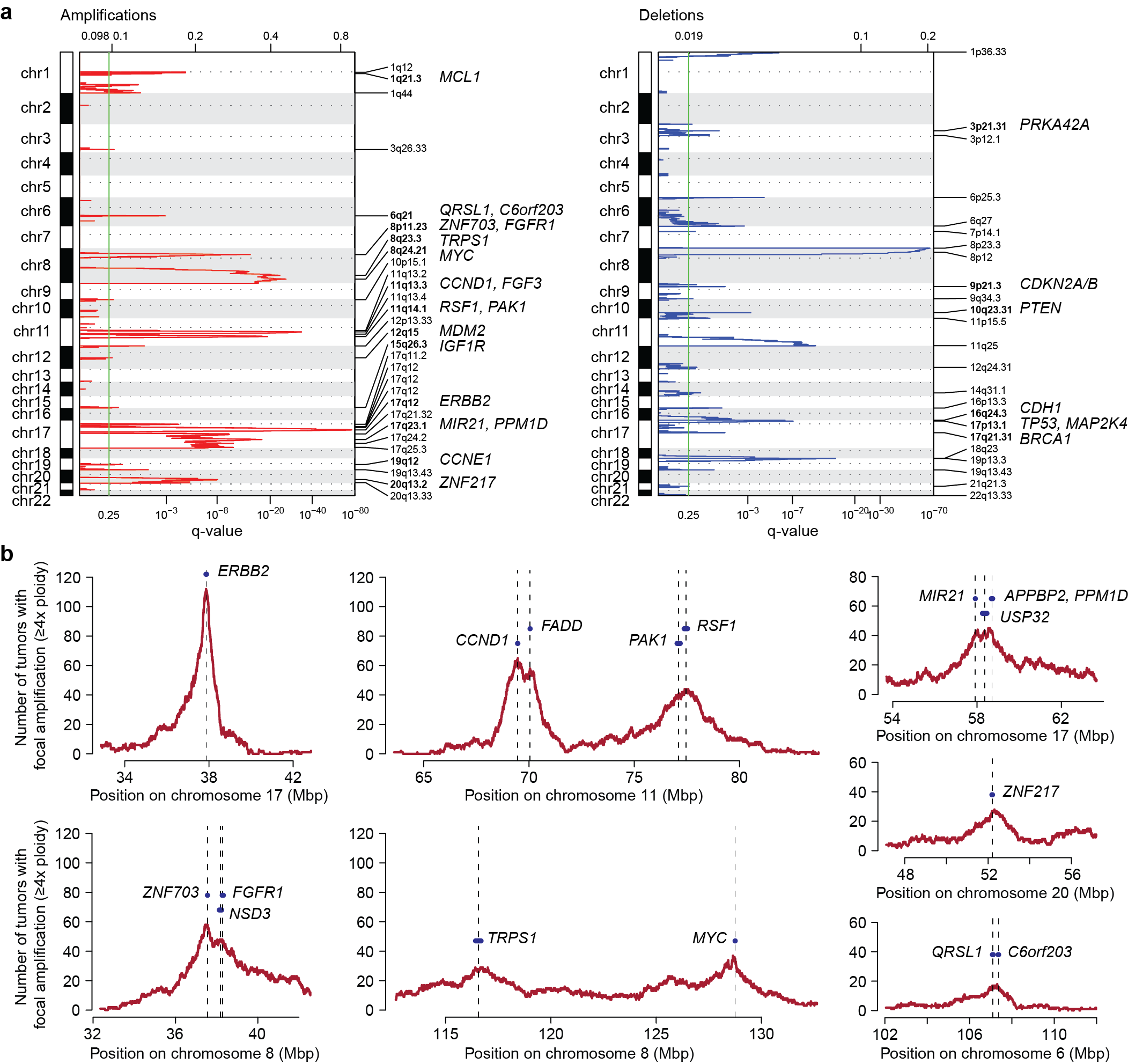


**Supplementary Fig. 2. Copy number peaks in 780 breast cancers.**

**a,** Amplification and deletion scores in GISTIC analysis. The top x axis indicates G-scores, a metric considering the amplitude and the frequency of the peaks, and the bottom x axis indicates q-values. **b,** Nine most recurrent amplification peaks in 780 breast cancers. Annotated genes are at the top of the peaks.

We found that some amplified regions have only one peak (e.g., *ERBB2*), but others have multiple peaks. For example, the prominent peak region on 11q13.3 has ‘double peaks’, of which the higher peak encompasses *CCND1*, and the lower peak targets a less-known gene in breast cancer, *FADD*. The 17q23 amplicon, which has been frequently observed in hormone receptor-positive breast cancers, has a broad copy-number elevation covering a megabase region (57.9-58.9 Mbp) with triple peaks. The highest peak encompasses an uncharacterized gene, *APPBP2*. This gene is in the neighborhood of *PPM1D*, an oncogenic protein phosphatase frequently altered in myeloid malignancies and several other tumor types. The other two peaks include *MIR21* and surrounding two large genes (*VMP1* and *TUBD1*) and *USP32*. The 8p11.23 peak region also showed the double peaks pattern. The higher peak has *ZNF703*, and the lower peak harbors the other two putative oncogenes, *NSD3* and *FGFR1*. Some target genes of the focal amplicons were largely unknown for their role in breast cancer. For example, 6q21 amplicon harbors several genes, including *QRSL1*, *MTRES1*, and *BEND3*. Some of these genes (*RSF1*, *PAK1*, and *TRPS1*) were suggested as putative breast cancer oncogenes in prior publications^70-72^.


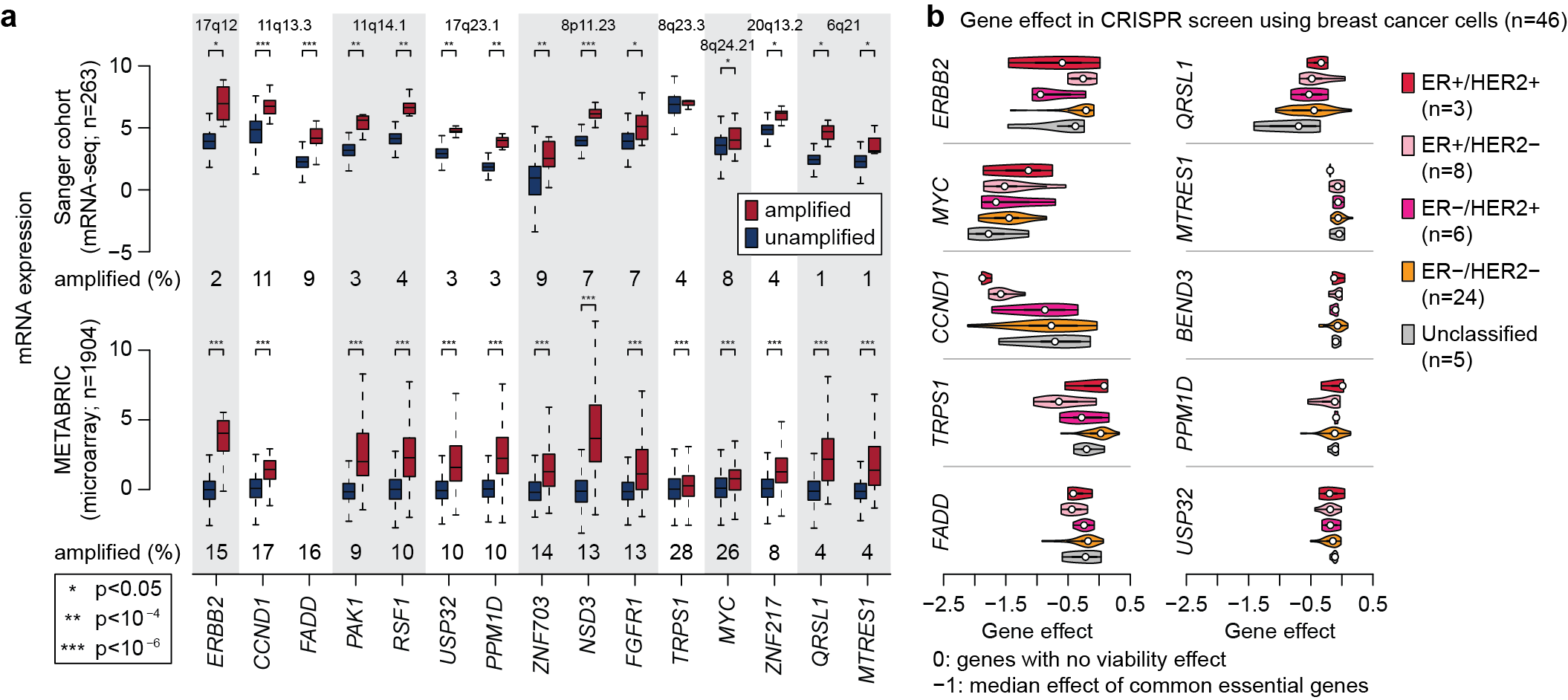


**Supplementary Fig. 3. RNA expression and dependency of the amplified genes.**

**a.** RNA expression of the 15 frequently amplified genes in the Sanger and the METABRIC cohorts. Statistical comparison was made by two-sided, two-sample *t* test. **b,** Knockout effect of frequently amplified genes in breast cancer cell lines in the DepMap project. Cell lines are classified by the ER and the HER2 expression. Gene effect score is used as the readout for cellular dependence on the given gene.

Next, we integrated the RNA sequencing information from Nik-Zainal *et al*.^15^. A total of 263 breast cancers with WGS were available with their paired RNA sequencing data. Using these tumors, we studied the RNA expression of the frequently amplified genes. We validated the findings in the METABRIC cohort^14^ using their diploid samples. Most genes showed robust RNA overexpression when focally amplified (**Supplementary Fig. 3a**). This tendency was similar between the Nik-Zainal *et al*. and METABRIC cohorts. Notable exceptions were *TRPS1* and *MYC*, whose RNA expression ranges largely overlapped between the amplified and unamplified tumors in both cohorts. However, both genes demonstrated their essentiality in the CRISPR screen (**Supplementary Fig. 3b**).

To study the functional importance of the amplified genes, we integrated CRISPR screen data from the DepMap project^24^ (**Supplementary Fig. 3b**). This dataset reproduced the functional dependence of known oncogenes in the relevant molecular context. For example, knockout of *ERBB2* showed selective cytotoxicity in HER2+ cell lines, in contrast to the modest effects in HER2− cell lines. Similarly, knockout of *CCND1* showed more profound cytotoxicity in ER+ cells than in ER− cells. CRISPR knockout of *FADD* showed moderate cytotoxicity in ER+ cells, the subtype where *FADD* was frequently amplified. *QRSL1* knockout also showed moderate cytotoxicity in breast cancer cells, unlike other co-amplified genes *MTRES1* and *BEND3*. Knockout of *TRPS1*, which was proposed as a transcriptional regulator in luminal breast cancers^72^, showed selective cytotoxicity in ER+/HER2− cells.

In summary, *FADD* and *QRSL1* could be previously unappreciated oncogenes in breast cancer, as these two genes were frequent targets of focal amplification and their amplification was associated with overexpression. Some breast cancer cell lines showed their modest dependency on these genes in the knockout screen, supporting their functional importance. Some of the previously proposed oncogenes in breast cancer, including *RSF1*, *PAK1*, *TRPS1*, *PPM1D*, *USP32*, *NSD3*, and *TRPS1*, were frequently targeted by focal amplification, indicating their active role in breast cancer.

**Mutational signature at the vicinity of the rearrangement breakpoints**

We compared the pattern of SNVs in the neighborhood of boundary SVs to those near the SVs within the amplicons (‘internal SVs’ in **Supplementary Fig. 4a-c**). Only 18% (44 out of 245) of the tumors with TB amplifications showed SNVs within the 100-bp window from the boundary translocations, but this fraction increased to 40% and 60% in 1-Kbp and 10-Kbp windows, respectively. Mutational signature analysis showed no difference in the mutational spectra between the neighborhoods of boundary vs. internal SVs, both showing significant contributions from SBS2 and SBS13, two APOBEC3-associated mutational signatures (**Supplementary Fig. 4b, c**). We also analyzed indels near the boundary vs. internal SVs (**Supplementary Fig. 4d**). However, the number of indels at the vicinity of the boundary translocations was too small for further analysis. For example, only 16 indels were found in the 1-Kbp window, 48 in 10-Kbp, and 187 in 100-Kbp. The 100-Kbp window analysis showed no noticeable differences between the spectra of indels close to the boundary SVs and the internal SVs (**Extended Data Fig. 4d**). Last, the boundary SVs showed a shorter microhomology at their break ends compared to what was observed for the internal SVs (1.39 vs. 1.70, p = 6.0 × 10^−8^ by two-sided, two-sample *t* test), consistent with our TB amplification model starting from the repair of DSBs by non-homologous end-joining.


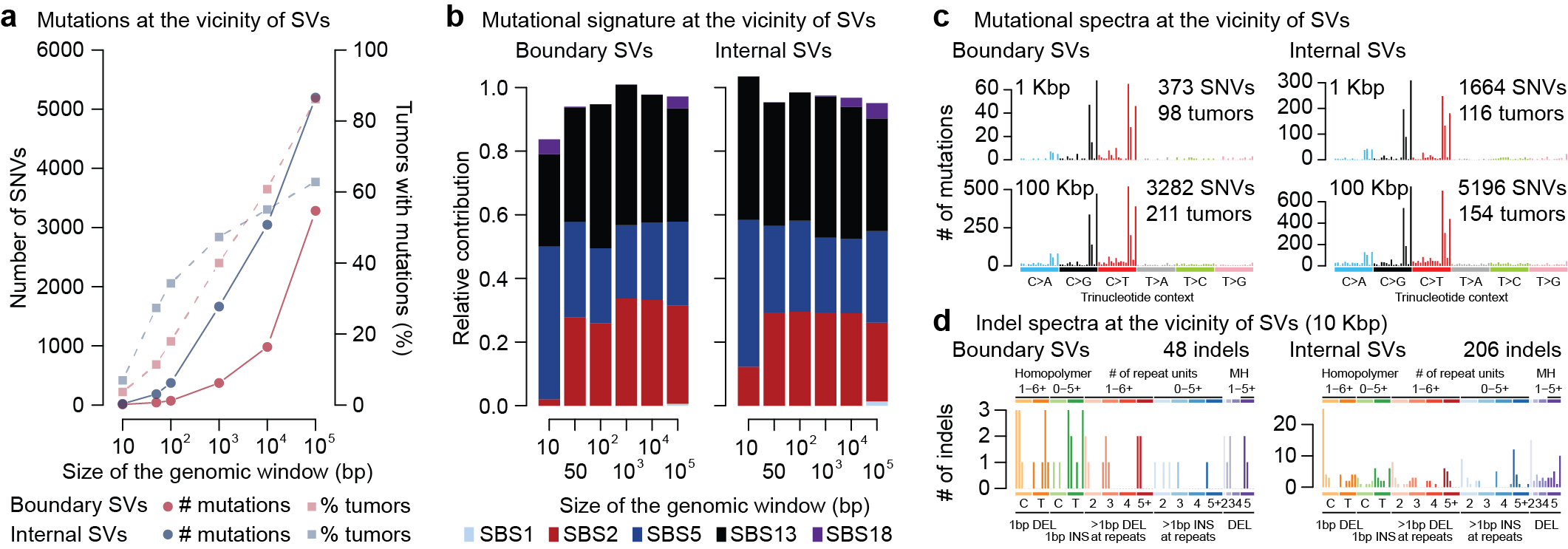


**Supplementary Fig. 4. Pattern of mutations near the TB amplification breakpoints.**

**a,** Number of SNVs at the vicinity of boundary translocations and internal SVs. Only the SNVs proximal to the SV breakpoints, considering their orientation, were used in this analysis. **b,** Mutational signatures, **c,** the 96-trinucleotide spectra of the SNVs, and **d,** the 83-type spectra of the indels at the vicinity of the TB amplification breakpoints.

**A male breast cancer case with translocation-bridge amplification**

Similar to what was observed in TCGA-A8-A08S (**Extended Data Fig. 3**), we found co-amplifications of multiple segments in 17q and 11q, involving *ERBB2*, *CCND1*, and *PAK1,* in a male breast cancer case, TCGA-A1-A0SM. The patient was a 77-year-old male with gynecomastia, a clinical sign of hyper-estrogenic state. We found two highly amplified translocations connecting the telomeric and the centromeric boundaries of the *ERBB2* and *PAK1* amplicons (blue and green translocations, respectively, in **Supplementary Fig. 5**). This reflects an ecDNA structure containing both oncogenes amplified up to >30 copies. The overall footprint is best explained by dicentric chromosome formation between 11q and 17q from the translocation at the telomeric borders of the co-amplicons (blue SV in **Supplementary Fig. 5**). LOH is observed in large areas of the both q arms. Heterozygosity of the telomeric portion of 17q was preserved because of its translocation to chromosome 1, which was likely a simultaneous event with the translocation forming the dicentric chromosome.


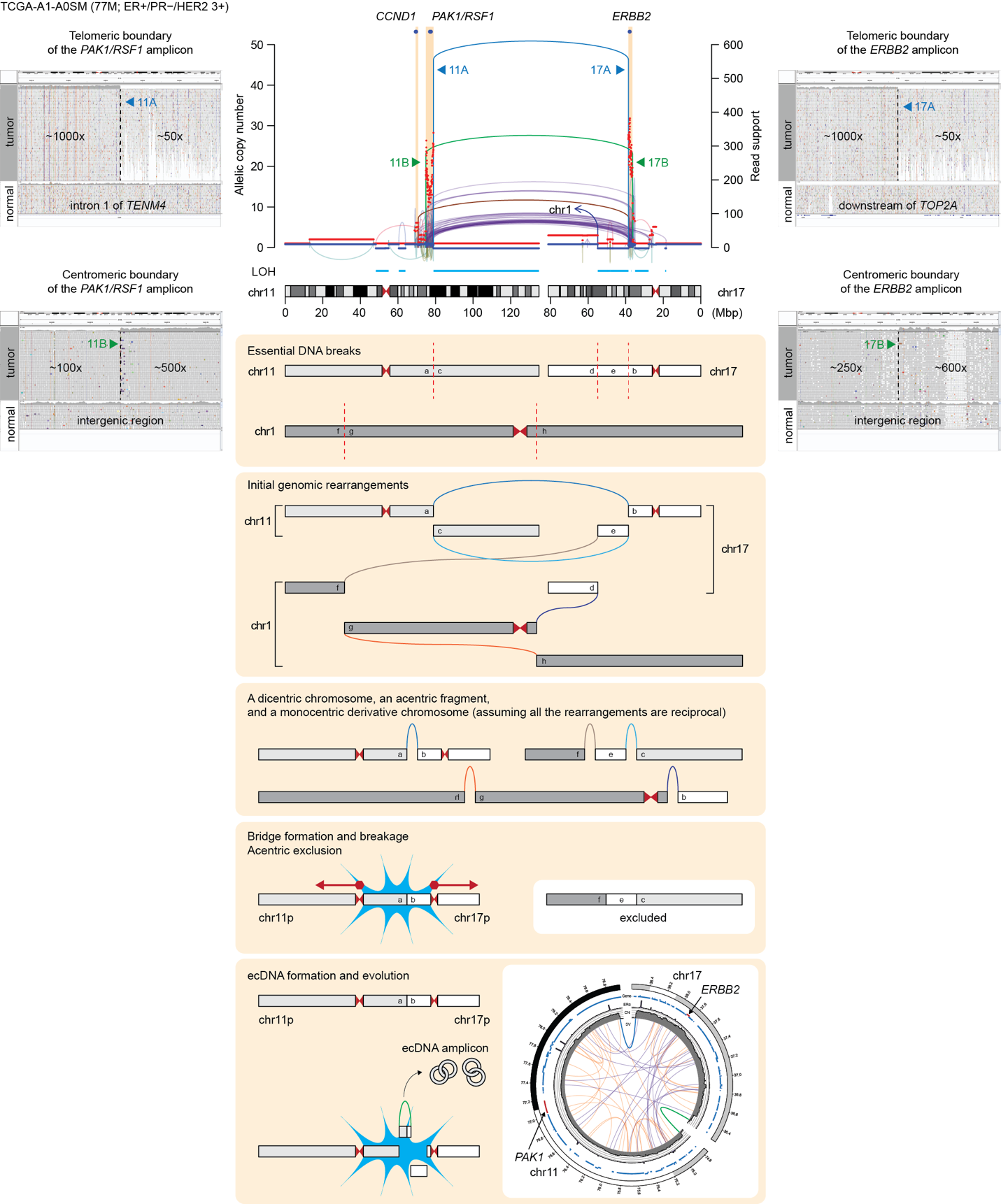


**Supplementary Fig. 5. Reconstruction of complex rearrangements in TCGA-A1-A0SM.**

Two SVs (blue and green colored lines) are particularly informative in inferring the anatomy of the dicentric chromosome bridge and its fragmentation. Two informative SVs are highlighted on the SV plot and their detailed structures are visualized by the Integrative Genomics Viewer.

**Fusion gene analysis**

Using LINX^56^, we identified 38,306 gene fusion events (including in-frame, out-of-frame, and exon-skipping events) in the 780 breast cancers. Surprisingly, the second most commonly fused gene in our cohort was *SHANK2* (233 events in 93 tumors; 12% of our cohort; **Supplementary Fig. 6a**), and the fourth was *TENM4* (178 events in 62 tumors; 8% of the cohort). These two genes are at the distal (telomeric) neighborhood of *CCND1* and *RSF1*/*PAK1*, respectively, frequently serving as the telomeric border of their amplicons (**Extended Data Fig. 8b** and **Supplementary Fig. 6b**). *BCAS3*, which is frequently observed in the right border or inside of the 17q23 amplicon, was ranked first by the number of fusion events in our analysis (284 events in 77 tumors; 10% of the cohort), but the number of tumors harboring its fusion was less than that of *SHANK2*.


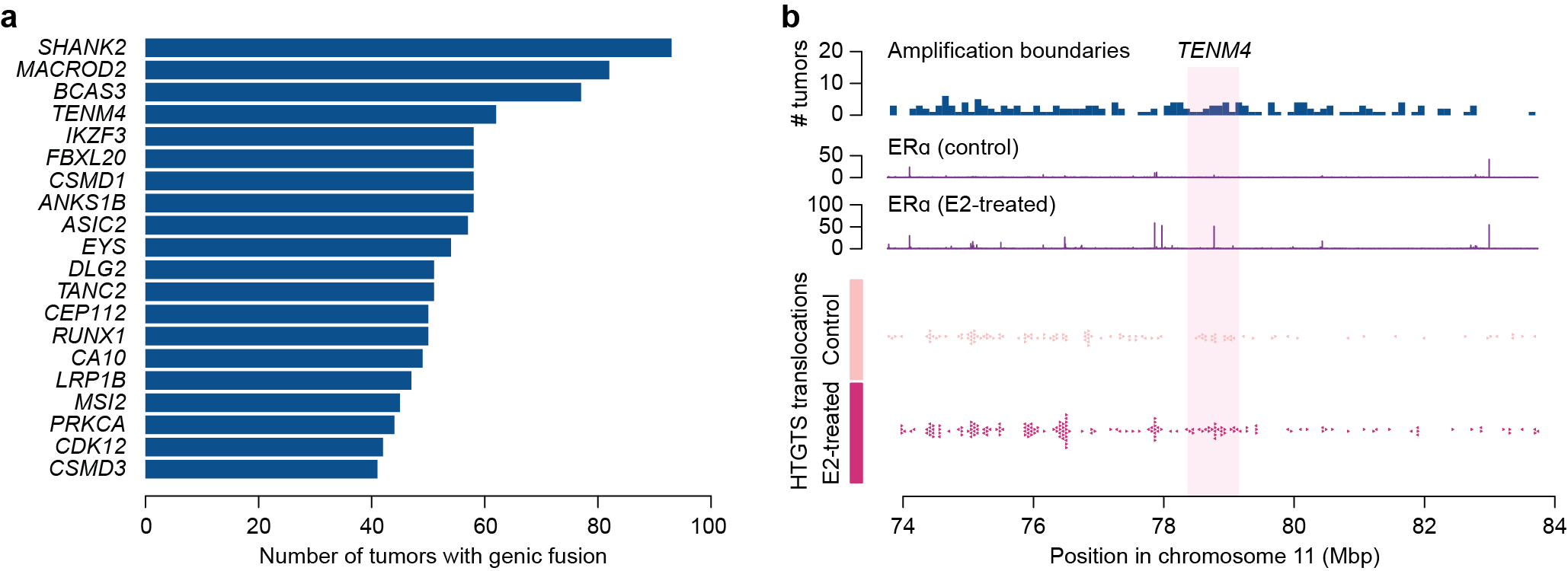


**Supplementary Fig. 6. Associations among structural variations, binding of ERɑ, and E2-induced translocations in HTGTS.**

**a,** Frequency of gene fusions in the 780 breast cancers by the LINX algorithm. Both intragenic and intergenic fusions are included in this analysis. **b,** Amplification boundaries around *TENM4* (upper panel; often serves as telomeric boundary of the *RSF1*/*PAK1* amplification), ERɑ binding with or without E2 treatment (middle panel), and the HTGTS translocations (lower panel).

Many fusions in these genes were predicted to produce in-frame sequence with the ligation partner genes (77% for *SHANK2* fusions, 69% for *TENM4* fusions, and 67% for *BCAS3* fusions). Accordingly, a recent RNA-seq analysis reported frequent in-frame fusion transcripts in these genes^73^. It appears that these genes are fragile in breast cancers, often providing the initial DSBs that could initiate TB amplification when they are rearranged to generate a dicentric chromosome. But, in addition, these genes can also be rearranged with the other genes more stably and generate their fused product. In summary, some genes that are frequently found at the boundary of TB amplifications, such as *SHANK2*, *TENM4*, and *BCAS3*, are also rearrangement hotspots and often produce gene fusions.

**Mechanisms of DNA breaks in *SHANK2* and *TENM4* loci**

Amplifications of *CCND1* and *RSF1*/*PAK1* were frequently initiated by the DNA breaks at *SHANK2* and *TENM4*, respectively. In our experiments with induced breaks in *RARA*, we observed increased HTGTS translocations in *SHANK2* and *TENM4* by the E2 treatment, consistent with the E2-induced, ERɑ-mediated fragility. However, the level of increase was not significantly different from the background increase by the E2 treatment (**Extended Data Fig. 8b** and **Supplementary Fig. 5b**). This was in contrast to the significantly increased translocations to *RARA* in the experiments with induced breaks in *SHANK2* (**Extended Data Fig. 8a**). In contrast to *RARA*, which is one of the canonical estrogen-responsive genes, *SHANK2* and *TENM4* are not known as estrogen-responsive genes^37^, although prominent E2-ERɑ peaks were observed in their introns. Canonical estrogen-responsive genes, such as *GREB1* and *ITPK1*, typically showed a substantial increase of translocation by E2 in our experiments (**Supplementary Fig. 7a**, **b**).

Given the role of R-loops in the formation of genomic rearrangements^35^, we examined if *SHANK2* or *TENM4* could have R-loop formation by the E2 treatment. In the DRIP-seq dataset from Stork *et al*. ^35^, we observed a mild degree of R-loop formation in *SHANK2* and a low degree in *TENM4*, both with no noticeable difference associated with E2 treatment (**Supplementary Fig. 7c**). In our LASSO regression model for the amplification boundary hotspots, R-loop was one of the significant variables with the boundary hotspots (p=0.029; **Fig. 3a**). This was significant in the ER+ subgroup but not in the ER− subgroup. In contrast, the E2-induced translocations in our HTGTS experiments were not significantly associated with R-loop (**Fig. 3f**), even though the duration of E2 treatment in our experiment was long enough to induce the R-loop formation in the late estrogen-responsive genes described by Stork *et al*.^35^ (R-loop formation was dramatically increased between 2 and 24 hours after the E2 treatment). Instead, the HTGTS translocations were strongly associated with both E2-ERɑ and topoisomerase 2B binding, which could be a more prevalent mechanism of E2-induced DSBs^34^. In summary, R-loop formation was associated with the amplification boundaries in breast cancer genomes but did not appear to be a dominant mechanism of E2-induced translocations in our experiment.


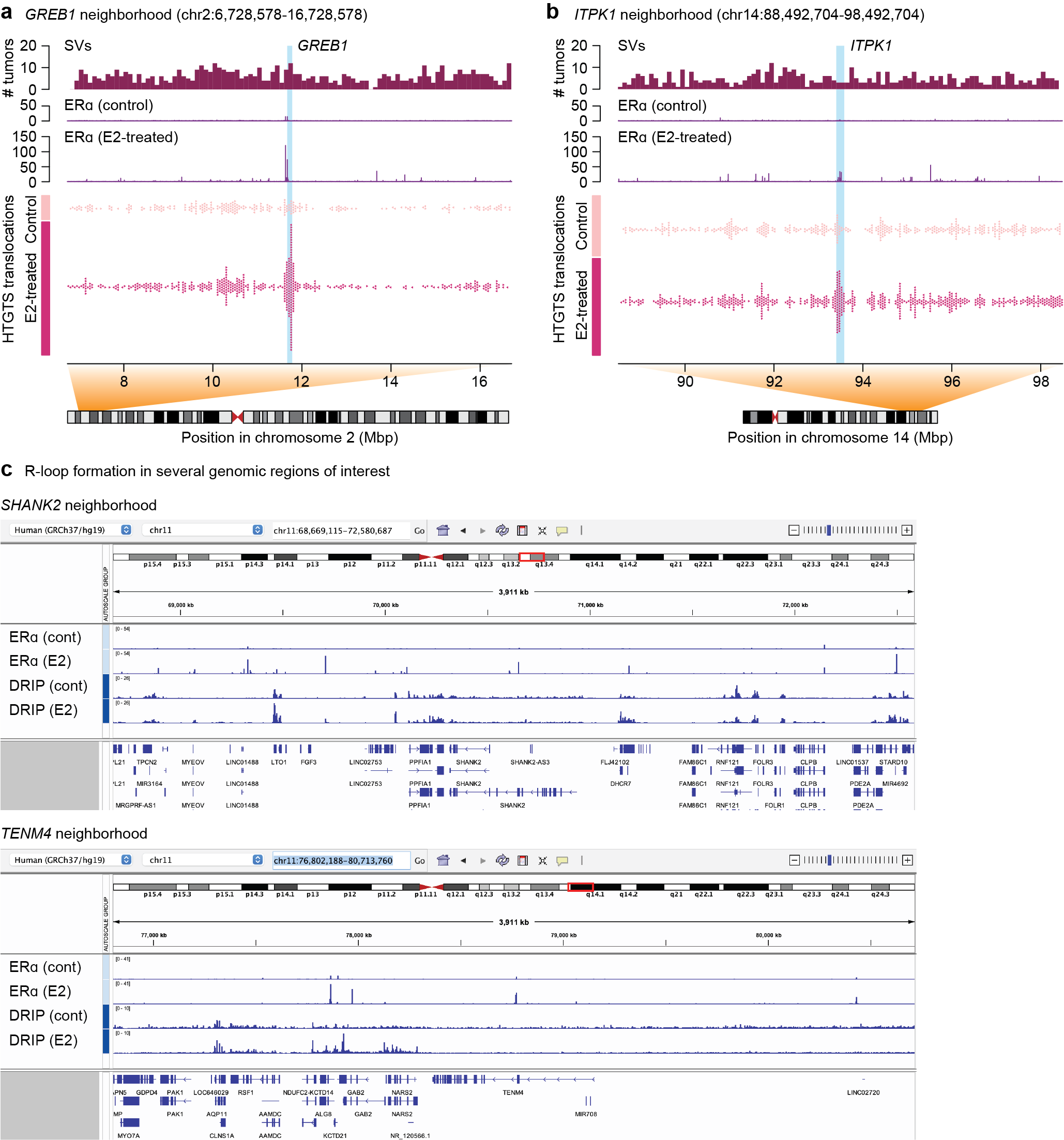


**Supplementary Fig. 7. ERɑ binding and R loop formation in the neighborhood of *SHANK2* and *TENM4*.**

Examples of two well-known ER target genes, **a,** *GREB1* and **b,** *ITPK1*, and the frequency of SVs in their neighborhood (upper panel), ERɑ binding profile (middle panel), and the HTGTS translocations (lower panel). **c,** DNA-RNA immunoprecipitation (DRIP) sequencing data was from Stork *et al*.^35^

**Role of physical proximity in early translocations**

Since physical proximity is a known contributory factor to the formation of translocations^74^, we explored if the chromosomal regions frequently involved in TB amplification were associated with frequent chromatin interactions. To compare the chromatin interactions in the E2-treated and control conditions, we obtained contact frequency information in MCF7 cells from Hsu *et al*. ^66^. We used the ratio of the arm-level contact frequencies in the E2-treated cells vs. the DMSO-treated cells (control) as an indicator for changes in chromatin contact by estrogen exposure. Then, we compared the changes in chromatin contact with the chromosome arm-level frequencies of translocations connecting the amplification boundaries. The arm pairs with recurrent TB amplifications (≥4) in our breast cancer cohort showed significantly higher interaction frequencies after the E2 treatment (**Supplementary Fig. 8a**), suggesting a potential role of estrogen in juxtaposing chromosomal segments, which could facilitate the formation of translocations.


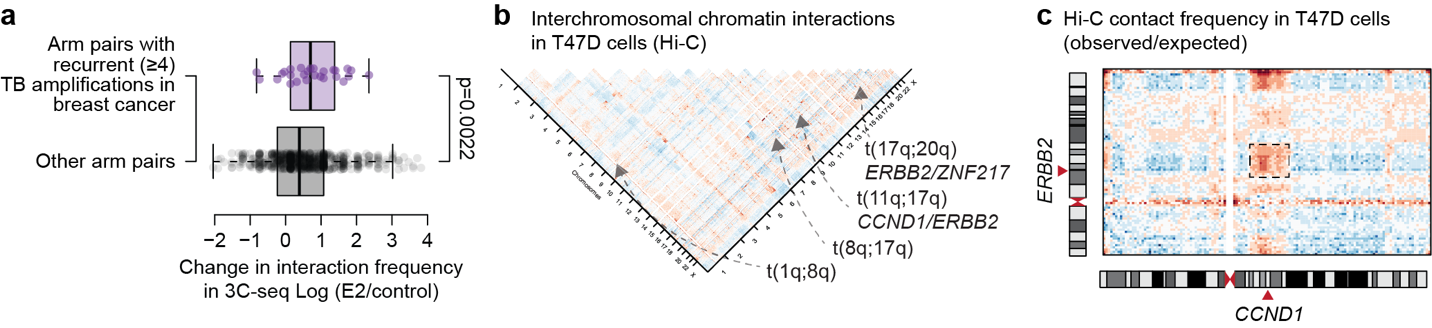


**Supplementary Fig. 8. Three-dimensional interaction analysis.**

**a,** Chromosome arm pairs with the frequent TB amplification events (≥4) in the 780 breast cancer genomes show significantly increased three-dimensional interactions in MCF-7 cells upon E2 treatment, compared to the other arm pairs. The highly recurrent (≥4) group includes 27 arm pairs and the others group included 791 pairs. Box plots indicate median (thick line), first and third quartiles (edges), and 1.5x of interquartile range (whiskers). Statistical comparison was made by rank sum test (one-sided). **b,** Inter-chromosomal chromatin interactions in T47D cells based on Hi-C. Observed/expected chromatin contact frequencies for genomic pairs between 2.5 Mbp-sized genomic bins were plotted. Translocation hotspots shown in **Fig. 1a** are annotated with arrows. **c,** A chromatin contact map (observed/expected) between chromosomes 11 and 17 in T47D cell line. Red indicates high contract frequencies and blue low frequencies. The dashed box indicates the region encompassing *ERBB2* and *CCND1*, exhibiting one of the highest contact frequencies among all pairs between chr11 and chr17.

We also asked if the hotspots of amplification boundaries were associated with high chromatin interaction frequencies in Hi-C data of T47D cell line without E2 treatment (**Supplementary Fig. 8b**). In this analysis, we found no tendency for increased contact frequencies among the recurrent amplification boundaries compared to all amplification boundaries or the background. As these Hi-C data was generated without the E2 treatment, it may reflect the baseline interaction rather than the estrogen-induced interactions. Nevertheless, some of the TB amplification hotspots, including the one involving *ERBB2* and *CCND1*, showed increased interaction frequencies (**Supplementary Fig. 8c**). According to a previous paper^75^, T47D does not have major inter-chromosomal translocations between the chromosomes of our interests, such as between chromosomes 8, 11, and 17.

**Supplementary Note References**

70 Sanchez-Garcia, F. *et al*. Integration of genomic data enables selective discovery of breast cancer drivers. *Cell* **159**, 1461-1475 (2014).

71 Arias-Romero, L. E., Villamar-Cruz, O., Huang, M., Hoeflich, K. P., and Chernoff, J. Pak1 kinase links ErbB2 to β-catenin in transformation of breast epithelial cells. *Cancer Res* **73**, 3671-3682 (2013).

72 Witwicki, R. M. *et al*. TRPS1 is a lineage-specific transcriptional dependency in breast cancer. *Cell Rep* **25**, 1255-1267 (2018).

73 Hoogstrate, Y. *et al*. Fusion transcripts and their genomic breakpoints in polyadenylated and ribosomal RNA-minus RNA sequencing data. *Gigascience* **10**, giab080 (2021).

74 Zhang, Y. *et al*. Spatial organizatio of the mouse genome and its role in recurrent chromosomal translocations. *Cell* **148**, 908-921 (2012).

75 Rondon-Lagos, M. *et al.* Unraveling the chromosome 17 patterns of FISH in interphase nuclei: an in-depth analysis of the HER2 amplicon and chromosome 17 centromere by karyotyping, FISH and M-FISH in breast cancer cells. *BMC Cancer* **14**, 922 (2014).
