# Supplementary material for: ERα-associated translocations underlie oncogene amplifications in breast cancer
Source: Nature. 2023 May 17;618(7967):1024–32. doi: 10.1038/s41586-023-06057-w (PMC10307628; doi:10.1038/s41586-023-06057-w)
Supplement: Supplementary file 2 — Reporting Summary [file 41586_2023_6057_MOESM2_ESM.pdf]

nature portfolio

Corresponding author(s): Jake J. Lee and Peter J. Park

Last updated by author(s): Mar 28, 2023

Reporting Summary

Nature Portfolio wishes to improve the reproducibility of the work that we publish. This form provides structure for consistency and transparency in reporting. For further information on Nature Portfolio policies, see our Editorial Policies and the Editorial Policy Checklist.

Statistics

For all statistical analyses, confirm that the following items are present in the figure legend, table legend, main text, or Methods section.

n/a	Confirmed	
☐	☒	The exact sample size (*n*) for each experimental group/condition, given as a discrete number and unit of measurement
☐	☒	A statement on whether measurements were taken from distinct samples or whether the same sample was measured repeatedly
☐	☒	The statistical test(s) used AND whether they are one- or two-sided<br>*Only common tests should be described solely by name; describe more complex techniques in the Methods section.*
☐	☒	A description of all covariates tested
☐	☒	A description of any assumptions or corrections, such as tests of normality and adjustment for multiple comparisons
☐	☒	A full description of the statistical parameters including central tendency (e.g. means) or other basic estimates (e.g. regression coefficient) AND variation (e.g. standard deviation) or associated estimates of uncertainty (e.g. confidence intervals)
☐	☒	For null hypothesis testing, the test statistic (e.g. *F*, *t*, *r*) with confidence intervals, effect sizes, degrees of freedom and *P* value noted<br>*Give P values as exact values whenever suitable.*
☒	☐	For Bayesian analysis, information on the choice of priors and Markov chain Monte Carlo settings
☒	☐	For hierarchical and complex designs, identification of the appropriate level for tests and full reporting of outcomes
☐	☒	Estimates of effect sizes (e.g. Cohen's *d*, Pearson's *r*), indicating how they were calculated

Our web collection on statistics for biologists contains articles on many of the points above.

Software and code

Policy information about availability of computer code

Data collection

Data was downloaded and extracted with EGA Download Client V3 (available at https://github.com/EGA-archive/ega-download-client).

Data analysis

List of softwares:
- R (version 4.1.1): https://www.r-project.org
- BWA MEM (version 0.7.15): https://github.com/lh3/bwa
- Samtools (version 1.3.1): https://github.com/samtools/samtools
- Bazam (version 1.0.1): https://github.com/ssadedin/bazam
- Picard (version 2.8.0): http://broadinstitute.github.io/picard
- PURPLE (version 2.54): https://github.com/hartwigmedical/hmftools/blob/master/purple
- COBALT (version 1.11): https://github.com/hartwigmedical/hmftools/blob/master/cobalt
- AMBER (version 3.5): https://github.com/hartwigmedical/hmftools/blob/master/amber
- GRIDSS2 (version 2.12.0): https://github.com/PapenfussLab/gridss
- RepeatMasker (version 4.1.2-p1): https://github.com/rmhubley/RepeatMasker
- Kraken2 (version 2.1.2): https://github.com/DerrickWood/kraken2
- GRIPSS (version 1.9): https://github.com/hartwigmedical/hmftools/blob/master/gripss
- LINX (version 1.15): https://github.com/hartwigmedical/hmftools/blob/master/linx
- SAGE (version 2.8): https://github.com/hartwigmedical/hmftools/blob/master/sage
- CHORD (version 2.00): https://github.com/UMCUGenetics/CHORD
- xTea (version 0.1.6): https://github.com/parklab/xTea
- SigProfilerMatrixGenerator (version 0.1.0): https://github.com/AlexandrovLab/SigProfilerMatrixGenerator
- MuSiCal (version 1.0.0-beta): https://github.com/parklab/MuSiCal

- MutationTimeR (version 1.00.2): https://github.com/gerstung-lab/MutationTimeR
- MACS (version 2): https://hbctraining.github.io/Intro-to-ChIPseq/lessons/05_peak_calling_macs.html
- Bowtie (version 1.2.2): https://github.com/BenLangmead/bowtie
- GSEA (version 4.2.3): https://www.gsea-msigdb.org/gsea
- GEAT (version 0.1): https://github.com/geatools/GEAT

Custom code is available (also stated in the manuscript): https://github.com/parklab/focal-amplification
No commercial software is used.

For manuscripts utilizing custom algorithms or software that are central to the research but not yet described in published literature, software must be made available to editors and reviewers. We strongly encourage code deposition in a community repository (e.g. GitHub). See the Nature Portfolio guidelines for submitting code & software for further information.

Data

Policy information about availability of data

All manuscripts must include a data availability statement. This statement should provide the following information, where applicable:

- Accession codes, unique identifiers, or web links for publicly available datasets
- A description of any restrictions on data availability
- For clinical datasets or third party data, please ensure that the statement adheres to our policy

WGS datasets generated through ICGC or PCAWG consortium are available at the ICGC Data Portal (http://dcc.icgc.org) with download instructions and links available in the downloading PCAWG data section (https://docs.icgc.org/pcawg/data/). For 72 tumors in French study by Ferrari et al., we downloaded the BAM files from European Genome-phenome Archive (EGA; https://www.ebi.ac.uk/ega/) under the accession number of EGAS00001001431. BAM or CRAM files from the Sanger 560 breast cancer project were downloaded from EGA under the accession number of EGAD00001001334, EGAD00001001335, EGAD00001001336, EGAD00001001338, and EGAD00001001322, those from the British Columbia study were under EGAS00001001159 (more detailed sample-by-sample accession numbers are available in Table S4 in the published paper), and those from the Yale inflammatory breast cancer project were under EGAS00001004117. HTGTS dataset is available in Gene Expression Omnibus (GEO) under the accession number of GSE227369. MSigDB gene set collections are available at GSEA website (http://www.gsea-msigdb.org/gsea/downloads.jsp). Epigenomic datasets are also publicly available at Gene Expression Omnibus (GEO; http://www.ncbi.nlm.nih.gov/geo), 4D Nucleome Data Portal (http://data.4dnucleome.org), and other repositories under accession numbers provided in Supplementary Table 3. Somatic variant calls, including SNVs, indels, SVs, and allelic copy number information for 780 breast cancer cases are available at the Park lab website (http://compbio.med.harvard.edu/TBAmplification/).

Human research participants

Policy information about studies involving human research participants and Sex and Gender in Research.

Reporting on sex and gender	This is a meta-analysis study that we do not directly collect information from participants. Instead, we collected sex information from five published studies. Because breast cancer predominantly affects female, and the biological mechanism that we describe in the paper is associated with female endocrine physiology, we focused on biological sex in our study.
Population characteristics	This study primarily describes 780 patients with breast cancer. This is a meta-analysis of five published studies based on whole-genome sequencing, and the details of each study is available in "Patient cohort" section of the Methods. Clinicopathologic characteristics of the patients are described in Extended Data Fig. 1.
Recruitment	This study is a meta-analysis without direct recruitment of participants. Whole-genome sequencing datasets were obtained from public repositories (accession numbers available in the data availability section of the Methods).
Ethics oversight	The institutional review board of the Harvard Faculty of Medicine approved this study (IRB18-0151). Individual studies complied required ethical guidelines per published manuscripts.

Note that full information on the approval of the study protocol must also be provided in the manuscript.

Field-specific reporting

Please select the one below that is the best fit for your research. If you are not sure, read the appropriate sections before making your selection.

☒ Life sciences ☐ Behavioural & social sciences ☐ Ecological, evolutionary & environmental sciences

For a reference copy of the document with all sections, see nature.com/documents/nr-reporting-summary-flat.pdf

Life sciences study design

All studies must disclose on these points even when the disclosure is negative.

Sample size	For genome analysis, no sample size calculations were performed. Sample size was determined by the number of sequencing datasets available in each individual study. In total, breast cancer genomes from 780 patients were analyzed in this study. For in vitro experiments, n=3 biological replicate experiments were performed for reliability and feasibility for statistical analysis. Each biological replicate is defined as an independent cell cultures.

Data exclusions	We originally downloaded 787 cases. Five cases were excluded because they were sequenced from formalin-fixed paraffin-embedded tissues, and two cases were excluded due to the failure in quality assessment step in our bioinformatic analysis.
Replication	We performed high-throughput genome-wide translocation sequencing with three biological replicates per experiment. Result was concordant among the biological replicates. Details are available in Extended Data Fig. 7.
Randomization	No randomization was performed, given the descriptive nature of the study.
Blinding	No blinding was performed, given the descriptive nature of the study.

Reporting for specific materials, systems and methods

We require information from authors about some types of materials, experimental systems and methods used in many studies. Here, indicate whether each material, system or method listed is relevant to your study. If you are not sure if a list item applies to your research, read the appropriate section before selecting a response.

Materials & experimental systems

n/a	Involved in the study
☒	☐ Antibodies
☐	☒ Eukaryotic cell lines
☒	☐ Palaeontology and archaeology
☒	☐ Animals and other organisms
☒	☐ Clinical data
☒	☐ Dual use research of concern

Methods

n/a	Involved in the study
☒	☐ ChIP-seq
☒	☐ Flow cytometry
☒	☐ MRI-based neuroimaging

Eukaryotic cell lines

Policy information about cell lines and Sex and Gender in Research

Cell line source(s)	MCF7 (ATCC), T47D (ATCC), and 293FT (Invitrogen/ThermoFisher)
Authentication	The suppliers of these cell lines provide information on the generation, characteristics, and authentication of the cell line in its website (MCF7: https://www.atcc.org/products/htb-22; T47D: https://www.atcc.org/products/htb-133; 293FT: https://www.thermofisher.com/order/catalog/product/R70007). Cell lines authentication was performed using short tandem repeat (STR) by the supplier.
Mycoplasma contamination	The cells were tested negative for Mycoplasma contamination
Commonly misidentified lines (See ICLAC register)	None listed in the ICLAC register.
